# Bioluminescent RIPoptosome Assay for FADD/RIPK1 Interaction Based on Split Luciferase Assay in a Human Neuroblastoma Cell Line SH-SY5Y

**DOI:** 10.3390/bios13020297

**Published:** 2023-02-20

**Authors:** Parisa Ghanavatian, Hossein Salehi-Sedeh, Farangis Ataei, Saman Hosseinkhani

**Affiliations:** Department of Biochemistry, Faculty of Biological Sciences, Tarbiat Modares University, Tehran 14115-175, Iran

**Keywords:** split luciferase, FADD/RIPK1 interaction, RIPoptosome, SH-SY5Y cells, necroptosis

## Abstract

Different programed cell death (PCD) modalities involve protein–protein interactions in large complexes. Tumor necrosis factor α (TNFα) stimulated assembly of receptor-interacting protein kinase 1 (RIPK1)/Fas-associated death domain (FADD) interaction forms Ripoptosome complex that may cause either apoptosis or necroptosis. The present study addresses the interaction of RIPK1 and FADD in TNFα signaling by fusion of C-terminal (CLuc) and N-terminal (NLuc) luciferase fragments to RIPK1-CLuc (R1C) or FADD-NLuc (FN) in a caspase 8 negative neuroblastic SH-SY5Y cell line, respectively. In addition, based on our findings, an RIPK1 mutant (R1C K612R) had less interaction with FN, resulting in increasing cell viability. Moreover, presence of a caspase inhibitor (zVAD.fmk) increases luciferase activity compared to Smac mimetic BV6 (B), TNFα -induced (T) and non-induced cell. Furthermore, etoposide decreased luciferase activity, but dexamethasone was not effective in SH-SY5Y. This reporter assay might be used to evaluate basic aspects of this interaction as well as for screening of necroptosis and apoptosis targeting drugs with potential therapeutic application.

## 1. Introduction

Programed cell death (PCD) is a regulated cellular suicide through events inside a cell, promoting cell death through protein–protein interactions in supramolecular complexes that conduct the cell fate toward either survival or death [1]. Several types of PCD have been discovered in the past decades including autophagy, necroptosis, ferroptosis, apoptosis and pyroptosis [2]. Analysis of different PCD pathways provides evidence relating to disorders and the discovery of suitable drugs for their protein targets [3,4].

The activation of death receptors including tumor necrosis factor (TNF) receptor 1 (TNFR1), Fas (CD95), DR3, TNF-related apoptosis-inducing ligand (TRAIL) receptors (DR4 [TRAILR1], DR5 [TRAILR2]), Toll-like receptors (TLRs) and DR6 through intracellular death domains (DDs) mediate the oligomeric signaling complexes by recruiting the adaptor proteins such as TNFR1-associated death domain (TRADD), Fas-associated death domain (FADD), caspases (caspase8/10) and kinases (receptor-interacting protein kinase 1/3 (RIPK1/3) [5,6,7]. TNFR1 is the most well-known signaling pathway, resulting in different cell death modalities mediated by complex I (pro-survival), complex IIa, IIb (RIPoptosome) and necrosome [8]. Complex I is composed of TNF receptor associated factor 2 (TRAF2), TRADD, RIPK1 and the E3 ligases inhibitor of apoptosis proteins (IAPs) and the linear ubiquitin assembly complex (LUBAC) which are controlled by other E3 ligases via a series of ubiquitination, resulting in the activation of NF-kB pathway and a subsequent increase in the expression of cFLIP, a protein inhibitor of caspase 8, leading to inflammation or survival [9].

Upon intensifying the apoptotic pathway, complex I shifts to apoptotic-inducing complex IIa consisting of TRADD-FADD-caspase8 and complex IIb containing FADD-RIPK1-RIPK3-caspase 8-cFLIP which is called RIPoptosome. RIPoptosome triggers extrinsic apoptosis by a variety of simulators, including stimulation of cell death receptors, deletion of IAPs (cellular IAP 1/2 (cIAP1/2) and X-linked IAP (XIAP)) and genotoxic stress with chemotherapeutics treatment (e.g., etoposides), leading to caspase 8 activation and executioner’s caspases such as 3/7. However, RIPoptosome formation can result in necroptosis including FADD-RIPK1-RIPK3-MLKL by caspase 8 inhibition or allergic inflammation mediated by IL-33 (a prototypical alarmin), causing rapid degradation of RIPK1 [10,11,12]. RIPK1 and FADD are the most important actors of this pathway promoting apoptosis or necroptosis based on the levels of caspase 8 [13,14].

FADD is a bipartite adaptor protein containing both Death effector domain (DED) and DD. RIPK1 also possesses DD at its C-terminus and binds to FADD via homotypic DD:DD interactions, and caspase 8 contains tandem DEDs that allow the recruitment of FADD via DED: DED interactions. The RIPK1-DD: FADD-DD complex forms the core part in the oligomeric platform of the RIPoptosome with 2-MD, while the FADD DED: caspase 8 DED interaction is responsible for caspase 8 recruitment [10,15]. So far, negative-stain electron microscopy, modeling, immunoblotting (caspase 8 IP) and gel filtration confirm the RIPoptosome platform [10,11,16]. Because of differences in cellular contents in various cell types, the understanding of cell death platforms has been rather complex. Therefore, developing useful reporters can support better detection of these pathways with the aim of better understanding and curing numerous diseases such as cancer and neurodegenerative diseases [17,18].

Currently, the progress in the molecular area has been highly effective in the discovery of some important interactions in PCD’s complexes; however, some conundrums yet remain to be elucidated. Consequently, many techniques have been developed based on protein-fragment complementation assay using luciferase-based biosensors [19] to detect protein–protein interactions in apoptosis, necroptosis, pyroptosis and autophagy [20,21,22,23,24] Development of bioluminescent reporters for involving protein complexes in cell death enabled us to screen compounds against one of the large protein complexes in different cell death modalities [25,26,27,28,29,30].

In this study, we describe the interaction between FADD and RIPK1 in the presence of effective inducers and inhibitors of TNFα signaling by generating reporters based on a split luciferase complementation assay in SH-SY5Y as a caspase 8 negative cell line. Additionally, the effect of zVAD.fmk, BV6 and TNFα signaling pathways in this interaction and its following outcome was investigated.

## 2. Materials and Methods

### 2.1. Materials and Cell Culture

Cells were treated with recombinant human TNFα (R&D system), zyloxycarbonyl- Val-Ala-Asp(O-Me) fluoromethylketone (zVAD.fmk (abbreviated to Z) (Bachem, Bubendorf, Switzerland; no. N-1510), Necrostatin-1 (Nec-1) (Calbiochem, San Diego, CA, USA; no. 480065), BV6 (B) (Invitrogen), etoposide (ETPO), bortezomib (BOR) and dexamethasone (DEXA). The following antibodies were used for Western blotting: primary antibodies diluted 1:10,000 consist of (Goat anti-mouse; Jackson Immuno Research) and (Goat anti-rabbit; Jackson Immuno Research). Mouse anti FADD (Enzo Life Sciences no. Q13158, 1:1000), rabbit anti-RIP1 antibody (Cell Signaling Technology, 1:1000, no.610459), rabbit anti caspase 3 (Cell Signaling Technology, 1:1000, no.9662) and rabbit polyclonal anti-XIAP (Cell Signaling, 2042). Human SH-SY5Y neuroblastoma cells (ATCC, Manassas, VA, USA, RRID: CVCL_0019) were cultured in DMEM (Gibco, Waltham, MA, USA) supplemented with 10% fetal bovine serum (FBS) and 1% penicillin, streptomycin (Gibco, Waltham, MA, USA) at 37 °C in a 5% CO_2_ incubator.

### 2.2. Reporter Constructs and Site Directed Mutagenesis

The constructs of mouse RIPK1 (mRIPK1), mouse FADD (mFADD) and ubiquitin (Ub) promotor were first inserted in pEntry vectors (gifted by VIB, Gent University). We first put the *SacI* and *SacII* restriction sites using Phusion High-Fidelity DNA polymerase (Finnzymes, Life technologies) on the pEntry3C vector, containing *att*L1 and *att*L2 sites and the pEntryR2L3 vector, containing *att*R2 and *att*L3 sites to introduce, respectively, to make them for cloning genes with the Gateway^®^ system (please see Supplementary Table S1 for nomenclature and a complete list of plasmids). NLuc (1–416 amino acids) and CLuc (395–550 amino acids) of the luciferase sequence originated from pGL3-Control Vector (Promega). PCR was performed using Forward and Reverse primers (Table S1). The sequences coding for NLuc or CLuc with the GS-rich linker were introduced by ligation in the pEntryR2L3 vector using *SacII* and *XhoI* restriction sites. The sequences coding for mRIPK1 and mFADD were introduced in the pEntry3C vector using the CloneEZ PCR cloning kit (GenScript) with, respectively, the *SacI* and *SalI* or *BamH1* and *XhoI* restriction sites. The sequences coding for the Ub promoter were introduced by ligation in the pEntryL4R1 vector using the *BamHI* and *XhoI* restriction sites. All used fragments were amplified by PCR as shown in Table S2 and Figure S1A.

The vectors were transformed into the MC1061 *E. coli* strain and cultured at 37 °C for 24 h. Positive clones were selected using LB plates containing kanamycin. After plasmid extraction from broth cultures, obtained clones were validated by double digestion as well as sequencing (Figure S1B).

**Figure 1:**
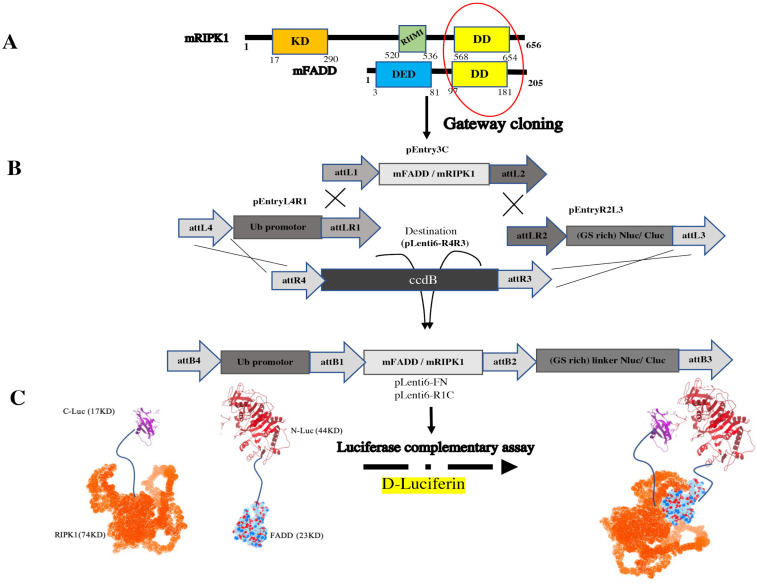
Strategy of generating FN and R1C. (**A**) The sequences of mFADD and mRIPK1 that bind to each other in DD homotypic interaction (marked with a red circle). (**B**) Cloning procedure of constructs was conducted by Gateway cloning. (**C**) Split luciferase enabled sensor for the detection of interaction between mRIPK1 and mFADD fused to CLuc and NLuc, respectively. The structures of RIPK1 (Uniprot: Q60855) and FADD (Uniprot: Q61160) are obtained from the AlphaFold Protein Structure Database (https://alphafold.ebi.ac.uk/, accessed on 20 July 2021).

These sequences were then recombined into the pLenti6-R4R3- puromycin destination vector using the LR gateway recombination system (Invitrogen). The Ub promotor and NLuc and CLuc sequences of luciferase was N and C-terminally fused to the coding sequence (mFADD-Nluc (FN) and mRIPK1-Cluc (R1C)) (Figure S3). Proper orientation of the Gateway cassette was confirmed by DNA sequencing. For validation of reporter, RIPK1 K612R was generated by QuikChange mutagenesis (Agilent Genomics) of R1C via PrimeSTAR^®^ HS DNA Polymerase (Takara). The vectors were transformed into the DH5α *E. coli* strain, cultured for 48 h at 28 °C on ampicillin plates. Positive clones were screened for the correct sequence by digestion and full-length sequencing after plasmid extraction from broth cultures. Final constructs are illustrated in Figure 1 and Figure S3.

### 2.3. Transient Transfection, Cellular Treatments and Extract Preparation

SH-SY5Y cells were seeded at 5 × 10^5^ cell/well in six-well plates and R1C and FN co-transfected 24 h later with a mix complex consisting branched polyethyleneimine (PEI, 25 KD). Reagents and 2 µg of each plasmid were added to each well [PEI/DNA ratio (*w*/*w*) = 3:1] in confluency of 70–90% [31]. The media were changed after 3 h with fresh media. After 18 h, cells were either prestimulated with zVAD.fmk (25 µM), Nec-1 (5 µM), BV6 (3 µM) alone or in the respective combinations for 1 h followed by stimulation by TNFα (100 ng/mL). Moreover, cells were treated with fresh media including BOR, ETPO and DEXA for 24 h and collected 48 h post-transfection. After treatment, cells were trypsinized from the plates and rinsed twice with ice-cold Phosphate-buffered saline (PBS) and cells lysed by hypotonic lysis buffer containing 20 mM HEPES-KOH (pH 7.6), 1.5 mM MgCl_2_, 10 mM KCl, 1 mM EDTA, 1 mM DTT, 100 mM sucrose and 1 mM PMSF, which could keep intact mitochondria. After three times freeze thaw, the insoluble material was removed by centrifugation for 10 min at 13,000× g and 4 °C. Supernatant was collected for our analysis, and total proteins were quantified by Bradford [32].

### 2.4. Western Blot Analysis

Supernatants by 12% SDS–PAGE were electrophoretically transferred onto a nitrocellulose membrane at 250 mA for 150 min and then blocked with 5% skim milk in a PBS buffer containing 0.1% Tween 20 (PBS-T) for 2 h. After blocking, the membrane was incubated in primary antibody diluted to 1:10,000 for overnight at 4 °C. After washing with PBS-T for 3 times, the membrane was placed into secondary antibody solution and was incubated with the membrane for 1 h at room temperature. After 3 times washing, the membrane was incubated for 5 min and exposed the blot using an Alpha Innotech Imager using enhanced chemiluminescence (ECL) reagents (Lumigen, Southfield, MI, USA).

### 2.5. Luciferase Activity Measurements and Cell Death Assays

For analysis of split luciferase complementation activity, cells were stimulated for 24 h. The cells were analyzed for cell death induction 48 h post transfection. For investigation of the effects of drugs, cells were treated with zVAD.fmk and TNFα and then ETPO, BOR and DEXA. Ten μL of cell lysate were added to 10 μL of substrate solution (10 mM MgSO4, 2 mM D-Luciferin potassium salt (Resem, Lijnden, The Netherlands), 4 mM ATP, 50 mM Tris–HCl, pH 7.8) in the Sirius tube Luminometer (Berthold Detection System, Germany), and luciferase complementary activity were reported as relative light unit (RLU). 

### 2.6. Caspases 3 Activity Measurement

To probe apoptosis induction, cells were cultured in 12-well plate (0.3 × 10^5^ per well) and harvested and lysed in hypotonic lysis buffer after the indicated time as mentioned. Ac-DEVD-AMC (Enzo) were the substrate for caspase 3 activity. First, 15 μL of cell extract were mixed with 100 μL of assay buffer containing 50 mM HEPES, 1 mM DTT, 5 mM EGTA, and 10 μM DEVD-AMC. An increase in AMC fluorescence (excitation at 360 nm, emission at 460 nm) was detected for up to 30 min. The slope from the linear part of each curve was normalized to the protein concentration of the lysate in each reaction and normalized to the protein concentration of the lysate.

### 2.7. Measurement of Reactive Oxygen Species

Intracellular accumulation of reactive oxygen species (ROS) was measured using the cell-permeant fluorescent probe, 2′-7′ dichlorofluorescin diacetate (DCFH-DA, sigma). Briefly, at selected times, cells were collected by trypsinization and washed with PBS and then incubated with a solution of DCFH-DA (10 μM) under the dark condition for 30 min at 37 °C. At the end of treatment period, the fluorescent intensity was measured by a microplate reader at the excitation/emission wavelengths of 480/530 nm. The ROS fold changes were normalized by cell number.

### 2.8. Statistical Analysis

GraphPad prism 8 software was used to analyze the data and construct statistical graphs. One- and two-way ANOVA tests were used to compare differences between treated groups and their paired controls, respectively. Differences in compared groups were considered statistically significant with *p* values lower than 0.05; * *p* ≤ 0.05; ** *p* ≤ 0.01; *** *p* ≤ 0.001; **** *p* ≤ 0.0001. All experiments were repeated at least three times, and the data are expressed as the mean ±SD from representative experiments.

## 3. Results

### 3.1. The pEntry and Final Constructs were Generated by Gateway Cloning

In order to create split luciferase-tagged proteins, different pEntry constructs were made. pEntry3C was prepared for mFADD and mRIPK1; pEntryR2L3 was prepared for CLuc; NLuc; and pEntryL4R1 was only made containing Ub promoter to have all final constructs under Ub promoter (Figure S3). Ub promoter prevents overexpression of the tagged proteins and keeps its level in the status of endogenous in contrast to CMV promoter [33].

After bacterial transformation of pEntry vectors in MC1061 *E. coli* and plasmid extraction, clones were evaluated using enzymatic digest. pEntry3C, pEnrtyR2L3 and pEnrtyL4R1 constructs were double digested, respectively, by *SalI* and *SacI*; *PvuII*; *SacII* and *XhoI*; *BamHI* and *XhoI* for 2 h at 37 °C. In Figure S1B, the double digested product is shown by gel electrophoresis.

To investigate the homotypic interaction among DDs of FADD and RIPK1, both proteins were tagged in C-terminal by NLuc or CLuc. Additionally, the flexibility for the accurate folding of the fused proteins was provided by 5 amino acid-linker (GGSGS) between the luciferase tag and the protein [34].

After bacterial transformation in DH5α *E. coli* strain, the plasmids were extracted after 48 h incubation at 28 °C for NLuc and CLuc constructs (Figure S2). Primary validation was conducted using restriction analysis by *EcoRV* for 2 h at 37 °C. The developed constructs are summarized in Table S1.

### 3.2. ZVAD.Fmk Trigger Luciferase Activity and Protein Expression in Caspase 8 Deficient SH-SY5Y Neuroblastoma Cells

**Figure 2:**
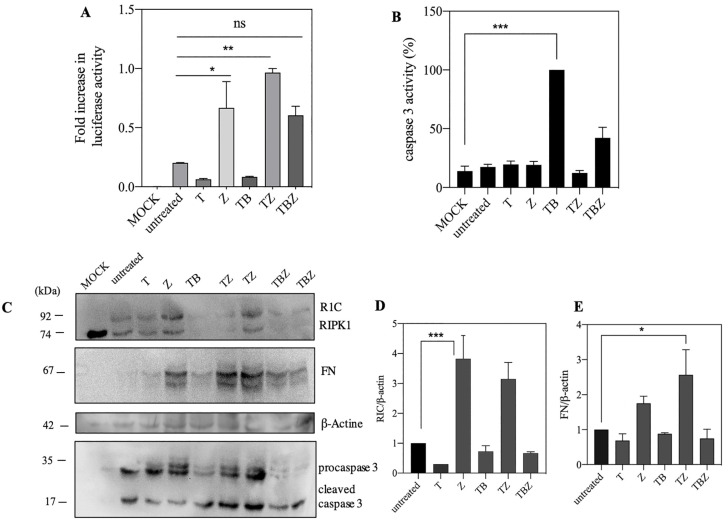
zVAD.fmk Promotes interaction between FADD and RIPK1. (**A**) SH-SY5Ycells were co-transfected with FN and R1C. Following transfection (24 h), cells were pre-treated by zVAD.fmk (25 μM), BV6 (3 μM) and after passing 1 h followed by stimulation with TNFα (100 ng/mL). ns, not significant; * *p* ≤ 0.05; ** *p* ≤ 0.01; *** *p* ≤ 0.001. (**B**) Caspase 3 activity applied with Ac-DEVD-AMC substrate of caspase 3 (**C**) expression of proteins was analyzed by western blotting. In the presence of TZ expression of RIPK1 and FADD were increased. However, TB did not increase protein expression (**D**,**E**) Relative expression levels of R1C and FN compared to β-actin were analyzed by ImageJ 1.53k, respectively.

The interaction between RIPK1 and FADD mediated extrinsic apoptosis pathway in presence of caspase 8 [35]. Interaction was performed in a caspase 8 deficient neuroblastoma cell line, which originally derived from a metastatic bone tumor biopsy and as a sub-line of the parental line SK-N-SH [36]. Previous data on SH-SY5Y showed that this cell line with low expression of RIPK3, MLKL and caspase 8 probably have a novel necroptosis-like type of cell death [37,38]. Thus, we selected this cell line to better understand the role of FADD/RIPK1 interaction in necroptosis or apoptosis in absence of caspase 8. To evaluate this protein–protein interaction, after 24 h of transfection with PEI, we pre-treated SHSY5Ycells with pan-caspase inhibitor, zVAD.fmk (Z) and BV6 as a bivalent Smac mimetic (B) for 2 h, followed by addition of TNFα (T) alone or combined (combination hereafter referred as TBZ). After 48 h, lysed cells were used for experimental data. Based on previous studies, unexpectedly, we could not find any change in FN and R1C interaction in SHSY5Y in the presence of B, but interestingly, Z increased interaction between them than untreated cell, as expressed by luciferase reconstitution activity (Figure 2A). Western blot displayed the increased expression of both R1C and FN in the presence of TZ and Z treatment, leading to the increase in luciferase activity (Figure 2C–E) in contrast to interaction with TB, which induced minimal interaction. However, based on caspase 3 activity, the highest activity was observed in TB treated whereas TZ treated cells indicated low level of apoptosis as displayed by caspase 3 activity (Figure 2B).

### 3.3. K612R Mutation in DD of RIPK1 Reduce FADD/RIPK1 Interaction

**Figure 3:**
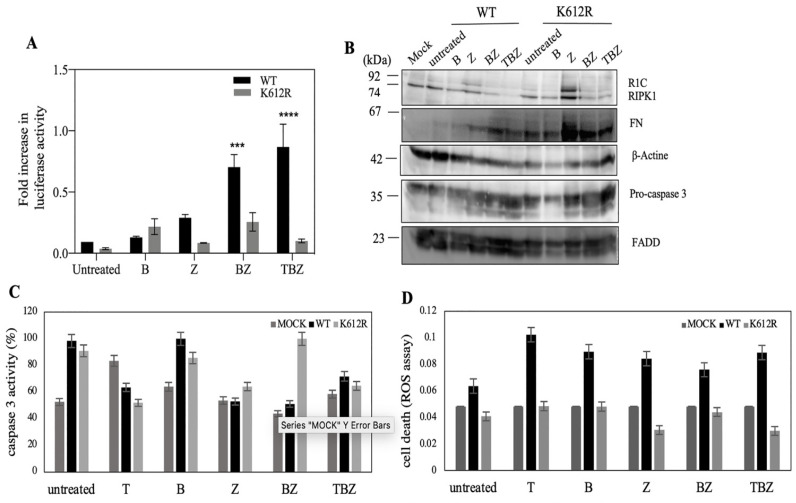
RIPK1 K612R revealed reducing luciferase activity compared to WT RIPK1. Co-transfected SHSY5Y cells with WT/RIPK1 K612R and FADD were pretreated with BV6 (5 µM) and zVAD.fmk (25 µM), and then with TNFα (100 ng/mL) for 18 h. (**A**) Luciferase activity as indicator of protein complementation; *** *p* ≤ 0.001; **** *p* ≤ 0.0001. (**B**) western blot of expressed proteins; (**C**) caspase-3 activity with Ac-DEVD-AMC substrate and (**D**) cell death was measured by ROS assay. Data are presented as mean ± SE; *n* = 3 of biologically independent samples. Two-way ANOVA with Bonferroni’s multiple comparison test.

A recent study with immunoblotting data showed that the K612R mutant in the mouse was a promising site in the ubiquitination effect on other DD-mediated interactions, especially FADD protein [39]. To approve FADD/RIPK1 interaction reporter assay, we made a K612R mutation in RIPK1 to confirm specificity of complementation. In comparison with luciferase activity of native, K612R showed lower activity (Figure 3A). Interestingly, zVAD.fmk had no effect on caspase 3 activity based on western blot data as well as cleavage of a fluorescent caspase-3 substrate (Figure 3B,D). These results support the idea that caspase 8 is not the main caspase in SH-SY5Y. In contrast to zVAD.fmk treatment, BV6 increased caspase 3 activity in R1C (WT and mutant) alone or combination with zVAD.fmk. Furthermore, ROS generation can happen in necroptosis [3]. ROS assay revealed reduction in cell death in K612R transfected cells compared to WT (Figure 3C). These findings show that R1C K612R with reduction of interaction with FADD probably decreased either apoptosis or necroptosis cell death.

### 3.4. Genotoxic Drugs such as Etoposide Decrease FADD/RIPK1 Interaction

**Figure 4:**
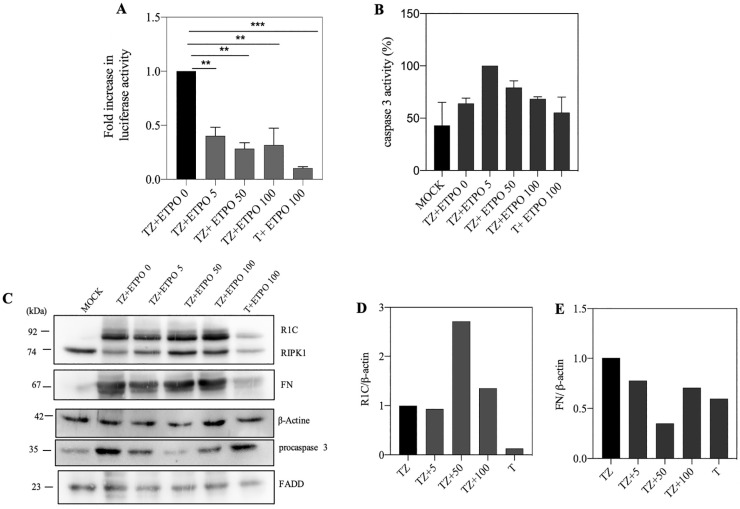
Etoposide effect on the binding of R1C and FN. (**A**) SH-SY5Y cells cotransfected with R1C and FN constructs by PEI. 24 h after transfection, cells treated with TZ or T and 2 h later, etoposide (5, 50 and 100 μM) were added. Split luciferase activity shows that interaction between R1C and FN decreased in presence of ETPO. ** *p* ≤ 0.01; *** *p* ≤ 0.001. (**B**) and Ac-DEVD-AMC activity increased with etoposide treatment. (**C**) Western blotting of recombinant and endogenous proteins (**D**,**E**) shows typical normalization of R1C and FN expression in western blot compared to β-actin.

Etoposide as an IAPs depletion compound for induction of cell death [40] can induce the binding of caspase 8 to RIPK1 and FADD only in cancer cells due to low expression of caspase 8 [10]. We examined the effects of etoposide in induction of cell death in SH-SY5Y; 5, 50 and 100 μM of etoposide were used which brought about with decrease in split luciferase complementary activity at 50 μM etoposide concentration (Figure 4A). Based on caspase 3 activity and western blot, the highest caspase 3 activity was observed at 5 μM, and the highest cleavage rate was observed at 50 μM of ETPO (Figure 4B,C). Normalized protein amounts showed that the highest amount of R1C and FN was observed at 50 μM of ETPO and in control, respectively (Figure 4D,E).

### 3.5. FN/R1C Interaction in Presence of as Bortezomib and Nec-1

**Figure 5:**
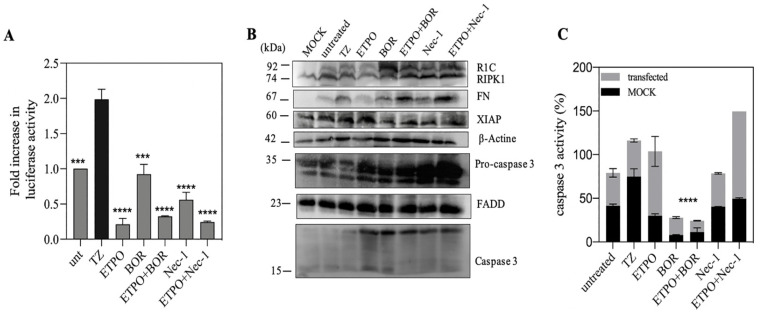
Etoposide effect in presence of bortezomib and Nec-1 based on luciferase activity. (**A**) After 24 h of co-transfection of FN and R1C with PEI. SH-SY5Y cells pre-treated with TZ and 2h later treated with ETPO (50 μM), BOR (40 nM) and Nec-1(5 μM). 18 h later cells were lysed for luciferase activity. (**B**) Western blot of recombinant and endogenous proteins. (**C**) Caspase 3 activity based on cleavage of Ac-DEVD-AMC. *** *p* ≤ 0.001; **** *p* ≤ 0.0001.

Since anti-apoptotic molecules such as in the IAP family are degraded by proteasome [40], increasing in anti-apoptotic molecules via some proteasome inhibitors such as bortezomib induces cell death that in combination with other drugs can improve therapies [10]. The kinase activity of RIPK1 requires the assembly of RIPoptosome so that using Nec-1, the RIPK1-targeted kinase inhibitor of necroptosis, can inhibit kinase domain activity and RIPoptosome formation [10]. To explore the role of etoposide in the presence of other treatments such as BOR and Nec-1, luciferase activity decreased in the presence of Nec-1 as same as ETPO; however the luciferase activity with bortezomib is more than both. Combination of ETPO + BOR and ETPO + Nec-1 decreased the luciferase activity compared to their alone application (Figure 5A). Data of caspase activity showed the lowest level in presence of BOR alone or its combination with ETPO (Figure 5B). On the other hand, using of Nec-1 with ETPO increased the caspase activity, and western blot also shows higher concentration of pro-caspase 3 in the presence of Nec-1 (Figure 5C). In addition, in the presence of BOR, expression of RIPK1 leads to the increase in luciferase activity. Some studies displayed that etoposide with triggering XIAP deletion promotes RIPoptosome formation [10,40]. In addition, western blot displayed that the amount of XIAP in ETPO compared with BOR is higher, as further confirmed with luciferase activity.

### 3.6. Dexamethasone Had no Effect on FN/R1C Interaction

**Figure 6:**
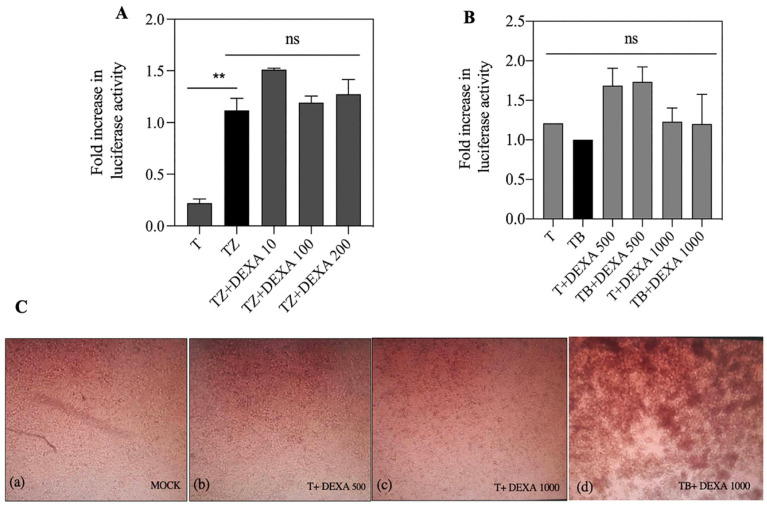
Transfected cells were treated for 18 h with indicated concentrations of dexamethasone in combination with zVAD.fmk (25 μM), BV6 (3 μM) and TNFα. SH-SY5Ycells were treated for 18 h with (**A**) TZ and (**B**) (TB) in the absence or presence of DEXA (concentrations indicated). (**C**) Morphology was visualized and photographed under a light microscope that cells become round and detached in medium after 18 h. Each graph shows the mean ± SD; *n* = 3 independent repeats. ns, not significant; ** *p* ≤ 0.01.

A previous study showed that dexamethasone has not any influence on caspase 8 interaction with FADD [10]. We treated SH-SY5Y cells with 10, 100 and 200 µM of DEXA in the presence of TZ (Figure 6A). Data showed that the FN/R1C interaction was not affected by DEXA treatment. In addition, synergistic combination of BV6 and dexamethasone has a significant effect on RIPoptosome formation [41]. So, we increased the concentration of DEXA to 500 and 1000 µM in the presence of BV6. Based on luciferase activity, no significant change in FN/R1C interaction was observed (Figure 6B). However, with microscopic imaging after 18 h of treatment, cell death was observed (Figure 6C).

## 4. Discussion

Split luciferase complementary assay has been used to keep track of the RIPoptosome complex formation between RIPK1 and FADD (Figure 1). Upon activation of TNFR1 in presence of a pan-caspase inhibitor (TZ) (Figure 2A), the increase in split luciferase activity indicates proper juxtaposition of complex subunits in presence of zVAD.fmk despite TNFα alone which might indicate resistance to TNFα in the absence of caspase 8. As well, lack of expression of caspase 8 has been documented as a resistance approach to TRAIL and chemotherapy in neuroblastoma cells [42]. However, to confirm right reporter interactions, mutation of critical involving residues has been implemented. There are many post-translational modifications (PTM) sites, such as ubiquitination, that are important in the TNFα signaling pathway [12]. RIPK1, as a mediator with different PTM sites, is more prone to ubiquitination in different sites especially in DD conduct TNFR1 pathway to cell death or survival [43]. K612 of RIPK1 has a pivotal role in its interaction with FADD [39]. The interaction of FADD/RIPK1 contributes to complex IIb which contains activated RIPK1, FADD and caspase 8 to mediate the activation of caspase 8 and apoptosis [44]. Mutation of K612 to R brought about with the loss of luciferase complementary activity presumably due to loss of proper interactions (Figure 3).

Several studies have shown that Fas or TNFR activation leads to necrotic cell death upon caspase inhibition in various cell lines [6,45]. Previous studies express that RIPK1/FADD have dual roles in extrinsic apoptosis and necroptosis [46]. Furthermore, the FADD and caspase 8 deficiency activates necroptosis [47]. Lake of expression of caspase 8 is frequent in the several kinds of tumor models such as lung carcinoma, neuroblastoma and hepatocellular carcinoma [48].

Our results showed zVAD.fmk treatment as a pan-caspase inhibitor not only increased FADD and RIPK1 expression but also elevated split luciferase complementary activity which is an indicator of higher RIPK1 and FADD interaction or at least their more proper proximity (Figure 2A). Some studies show that zVAD.fmk increases stability of the caspase 8 -RIPK1 complex most likely by protection from caspase-dependent apoptosis [13,49]. Likewise, over expression of RIPK1 leads to spontaneous formation of RIPoptosome [15]. However, Tenev et al., showed that treatment with zVAD.fmk did not affect RIPoptosome formation [10]. So, it could depend on variety of cell context in cell types. Furthermore, low expression of RIPK1 makes the cells resistant to cell death [13]. However, some data show that SHSY5Y cells do not have caspase 8 activity and expression [3]. Then, probably increasing in FADD/RIPK1 interaction induces cells towards necroptosis pathways.

Based on split luciferase complementary assay no significant change between R1C and FN interaction with BV6 treatment was observed (Figure 2A). Based on some studies, deletion of IAPs by tenoposide/etoposide and Smac mimetics such as BV6 promotes RIPK1/FADD/caspase8 interaction and spontaneous assembly of the Ripoptosome [41,50,51]. Moreover, cIAPs adjust the amount of RIPK1 in a cell type-dependent manner [50] and balance the intracellular levels of cIAPs and RIPK1 in RIPoptosome formation [13]. Lack of apoptosis extrinsic cell death could be attributed to either cells resistance against Smac mimetic compounds as reported earlier [51,52] or cells RIPK1 content. In spite of previous synergic reports on BV6 and Dexamethasone on hypersensitization of all cell lines to apoptosis [41], no significant changes in R1C and FN interaction were observed (Figure 4B and Figure 6A); based on split luciferase complementary assay, even more morphological cell death was observed.

Genotoxic drugs, such as Etoposide, can trigger cell death due to stabilization of the RIPoptosome complex (FADD/RIPK1 and caspase 8) [10]. A decrease in split luciferase complementary activity in the presence of etoposide may be due to a lack of caspase 8, as mentioned in the applied cell line. Therefore, it may be concluded that the RIPK1/FADD interaction may lead either to a necroptosis pathway or mitochondrial pathway of apoptosis, as indicated by higher caspase 3 activity. We co-treated the cells with bortezomib and Nec-1, and the results showed that co-treatment with bortezomib merely increased FN/R1C luciferase activity and decreased caspase-3 activity (Figure 5A,C), indicating the involvement of proteasomal degradation. Interestingly, the combination of ETOP + BOR decreased split luciferase activity without changes in caspase-3 activity (Figure 6A,C). These data are somehow in support of caspase 8 as an indispensable component of RIPoptosome complex.

## 5. Conclusions

In summary, according to the results presented in this manuscript, it can be concluded that FN/R1C bioluminescent reporter can be considered as an alternative approach to investigate basic aspect of RIPoptosome protein complex and to find suitable effective RIPoptosome disrupting or activating compounds.

## Data Availability

Not applicable.

## Supplementary Materials

The following supporting information can be downloaded at: https://www.mdpi.com/article/10.3390/bios13020297/s1, Figure S1. (**A**) agarose gel electrophoresis (1%) of PCR product of (mFADD and mRIPK1), (CLuc and NLuc) and Ub promotor for ligation in pEntry3C, pEntryR2L3 and pEntryL4R1 vectors, respectively. (**B**) double digestion of constructs for validation of cloning containing mFADD- pEntry3C (*BamHI and XhoI*) and mRIPK1- pEntry3C (*PvuII*), Nluc- pEnrtyR2L3 (*SalI and SacI*), CLuc- pEnrtyR2L3 (*SalI and SacI*) and Ub promoter- pEntryL4R1 (*BamHI and XhoI*); M (10 Kb); Figure S2. Digestion patterns of some final constructs using EcoRV, white marked clones are positive based on the band size (**A**) R1C; (**B**) FN; M (10 Kb); Figure S3. The Gateway cloning procedures for generating Luciferase-tagged proteins based on split luciferase assay using the PLenti6 destination vector. Firstly, the fragments encoding the Ub, mFADD, mRIPK1, NLuc and CLuc is inserted into a pEntry vector. Three pEntry vectors were described in this study: pEntry3C which used for mFADD and mRIPK1; pEntryL4R1 which used for Ub and pEntryR2L3 which used for luciferase fragments. In the end, the constructs in the pEntry vectors are recombined into pLenti6R4R3 vectors and generate (**A**) pLenti6-FN and (**B**) pLenti6-R1C; Table S1: Primers used for the amplification of tags; Table S2: PCR programs of the used genes.

## Abbreviations

B, BV6; BOR, Bortezomib; PBS, Phosphate-buffered saline; C-Luc, C-terminal of firefly luciferase; DEXA; Dexamethasone; DD, Death Domain; DED, Death effector domain; ETPO, Etoposide; FADD, Fas-associated death domain; FBS; fetal bovine serum; FN, FADD-Nluc; mRIPK1, Mouse RIPK1; mFADD, Mouse FADD; Nec-1, Necrostatin-1; NLuc, N-terminal of firefly luciferase; PCD, Programed cell death; RIPK1/3, Receptor-interacting protein kinase 1/3; R1C, RIPK1-Cluc; T, TNFα (Tumor necrosis factor α); TBZ, TNFα/BV6/zVAD.fmk; TNFR1, Tumor necrosis factor receptor 1; TRADD, TNFR1-associated death domain; Ub, Ubiquitin; PEI, polyethyleneimine; Z, zVAD.fmk (Benzyloxycarbonyl-Val-Ala-Asp-fluoromethylketone).
