# Supplementary material for: Bioluminescent RIPoptosome Assay for FADD/RIPK1 Interaction Based on Split Luciferase Assay in a Human Neuroblastoma Cell Line SH-SY5Y
Source: Biosensors (Basel). 2023 Feb 20;13(2):297. doi: 10.3390/bios13020297 (PMC9954477; doi:10.3390/bios13020297)
Supplement: Supplementary file 1 [file biosensors-13-00297-s001.zip › biosensors-2113034-supplementary.pdf]

Supplementary Materials

Bioluminescent RIPoptosome Assay for FADD/RIPK1 Interaction Based on Split Luciferase Assay in a Human Neuroblastoma Cell Line SH-SY5Y

Parisa Ghanavatian, Hossein Salehi-Sedeh, Farangis Ataei and Saman Hosseinkhani *

Table S1. Primers used for the amplification of tags.

Primer	Sequences [a]	Strategy
F-pEntry3C-*SacI*	CGGCCGCACTCGAGATAGAGCTCTAGACCCAGCTTTCTTGTAC '	Site directed mutagenesis
R-pEntry3C-*SacI*	GTACAAGAAAGCTGGGTCTAGAGCTCTATCTCGAGTGCGGCCG	Site directed mutagenesis
F-pEntryR2L3-*SacII*	CAACTTTCTT-GTACAAAGTGGCCGCGGTTAAAGGAACAAATTCAGTCG	Site directed mutagenesis
R-pEntryR2L3-*SacII*	CGACTGAATTGGTTCCTTTAACCGCGGCCACTTTGTACAA-GAAAGTTG	Site directed mutagenesis
F-mRIPK1-pEntry3C	AACCAATTCAGTCGACCATGCAACCAGACATGTCCTTGG	CloneEZ
R-mRIPK1-pEntry3C	GAAAGCTGGGTCTAGGCTCTGGCTGGCACG	CloneEZ
F-mFADD-pEntry3C	TTAGGATCCGCCATGGACCCATTCCTGGTGC	Ligation (*BamHI*)
R-mFADD-pEntry3C	GCGCTCGAGAGGGTGTTTCTGAGGAAGACACAG	Ligation (*XhoI)*
F-NLuc-linker-pEntryR2L3	TAACCGCGGAAGGTGGTTCTGGATCTATGGAAGACGCCA AAAACATAAAGAAAGGCCCG	Ligation (*SacII*)
R-NLuc-pEntryR2L3	ATACTCGAGCTAATCCTTGTCAATCAAGGCGTTGGTCGCTT CCG	Ligation (*XhoI)*
F-CLuc-linker-pEntryR2L3	TAACCGCGGAAGGTGGTTCTGGATCTATGATTATGTCCG GTTATGTAAACAATCCGGAAGCGACC	Ligation (*SacII*)
R-CLuc- pEntryR2L3	ATACTCGAGCTACACGGCGATCTTTCCGCCCTTCTTGGCC	Ligation (*XhoI*)
F-Ubiquitin promoter-pEntryL4R1	ATAGGGATCCGGCCTCGCGCCGGGTTTGGGC	Ligation (*BamHI*)
R-Ub promoter-pEntryL4R1	AGATCTCGAGGTCTAACAAAAAAGCC	Ligation (*XhoI)*
F-R1PK1 ^{K612R}	GGACTGAAAGAGAGAGTTTACCAAATGCTTC	Site directed mutagenesis
R-RIPK1 ^{K612R}	GAAGCATTTGGTAAACTCTCTCTTTCAGTCC	Site directed mutagenesis

[a] Underline sequence represents the restriction endonuclease site used for cloning and mutation sites.

Table S2. PCR programs of the used genes.

Gene	Plasmid	Initial denaturalization	Denaturalization	Annealing	Extension	Final extension
mRIPK1	pEntry3C	95℃ (5 min)	95℃ (45 Sec)	64℃ (30 Sec)	72℃ (150 Sec)	72℃ (7 Min)
mFADD	pEntry3C	95℃ (5 min)	95℃ (45 Sec)	60℃ (30 Sec)	72℃ (60 Sec)	72℃ (7 Min)
Ub promoter	pEntryL4R1	95℃ (5 Min)	95℃ (45 Sec)	65℃ (30 Sec)	72℃ (90 Sec)	72℃ (7 Min)
NLuc	pEntryR2L3	95℃ (5 Min)	95℃ (45 Sec)	65℃ (30 Sec)	72℃ (90 Sec)	72℃ (7 Min)
CLuc	pEntryR2L3	95℃ (5 Min)	95℃ (45 Sec)	66℃ (30 Sec)	72℃ (45 Sec)	72℃ (7 Min)
R1PK1 ^{K612R}	pLenti6-R1C $_{K612R}$	98 (3 Min)	98 (10 Sec)	55 (5 Sec)	72 (615 Sec)	72 (10 Min)

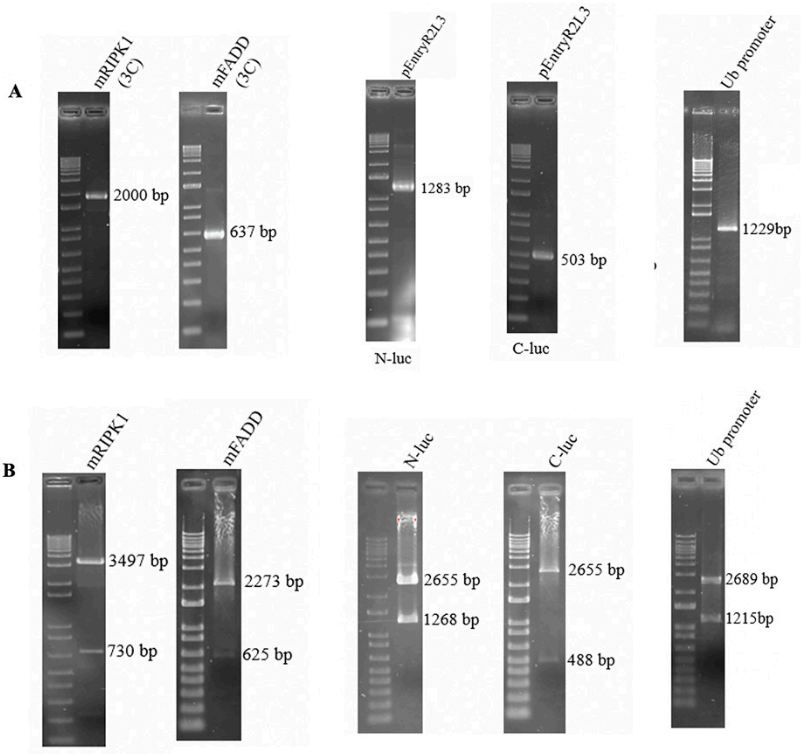

Figure S1. (**A**) agarose gel electrophoresis (1%) of PCR product of (mFADD and mRIPK1), (CLuc and NLuc) and Ub promotor for ligation in pEntry3C, pEntryR2L3 and pEntryL4R1 vectors, respectively. (**B**) double digestion of constructs for validation of cloning containing mFADD- pEntry3C (*BamHI* and *XhoI*) and mRIPK1- pEntry3C (*PvuII*), Nluc- pEnrtyR2L3 (*SalI* and *SacI*), CLuc-pEnrtyR2L3 (*SalI* and *SacI*) and Ub promoter- pEntryL4R1 (*BamHI* and *XhoI*); M (10 Kb).

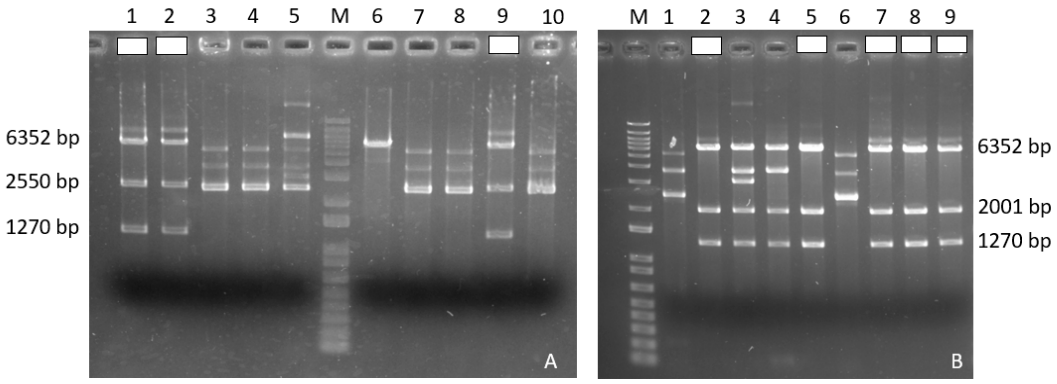

Figure S2. Digestion patterns of some final constructs using *EcoRV*, white marked clones are positive based on the band size (**A**) R1C; (**B**) FN; M (10 Kb).

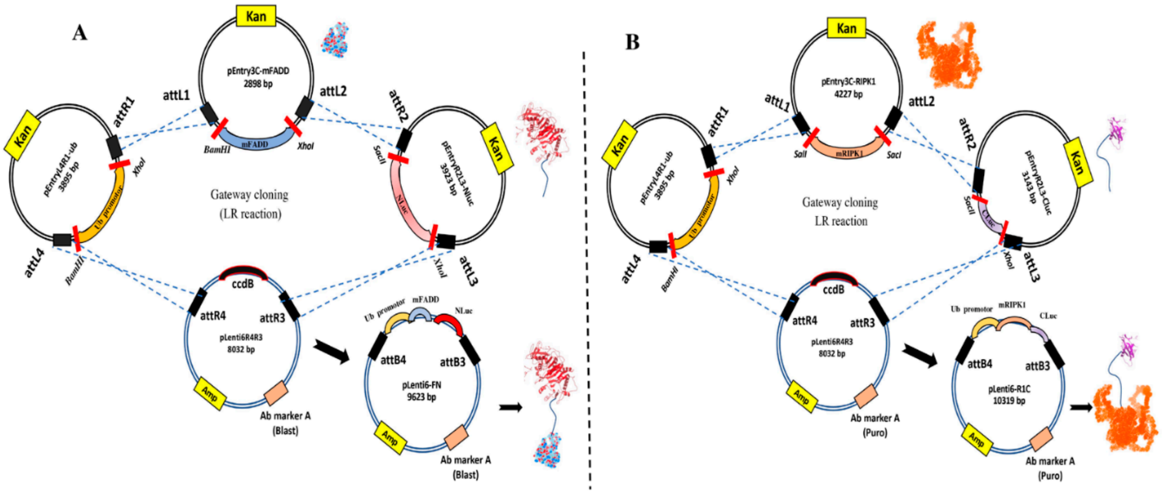

Figure S3. The Gateway cloning procedures for generating Luciferase-tagged proteins based on split luciferase assay using the PLenti6R4R3 destination vector. Firstly, the fragments encoding the Ub, mFADD, mRIPK1, NLuc and CLuc is inserted into an pEntry vector. Three pEntry vectors were described in this study: pEntry3C which used for mFADD and mRIPK1; pEntryL4R1 which used for Ub and pEntryR2L3 which used for luciferase fragments. In the end, the constructs in the pEntry vectors are recombined into pLenti6R4R3 vectors and generate (**A**) pLenti6-FN and (**B**) pLenti6-R1C.
